# Green Synthesized Copper-Oxide Nanoparticles Exhibit Antifungal Activity Against *Botrytis cinerea,* the Causal Agent of the Gray Mold Disease

**DOI:** 10.3390/antibiotics14111099

**Published:** 2025-11-02

**Authors:** Erisneida Campos-Jiménez, Karla Juarez-Moreno, Domingo Martínez-Soto, Alejandro Cabello-Pasini, Ernestina Castro-Longoria

**Affiliations:** 1 Department of Microbiology, Center for Scientific Research and Higher Education of Ensenada (CICESE), Carretera Tijuana-Ensenada 3918, Zona Playitas, Ensenada 22860, Baja California, Mexico; ecampos@cicese.edu.mx (E.C.-J.); dmartinez@cicese.edu.mx (D.M.-S.); 2 Center for Applied Physics and Advanced Technology, Universidad Nacional Autónoma de Mexico (UNAM), Juriquilla 76320, Queretaro, Mexico; kjuarez@fata.unam.mx; 3 Institute of Oceanological Research (IIO), Marine Botany Research Group, Universidad Autónoma de Baja California (UABC), Ensenada 22860, Baja California, Mexico; acabello@uabc.edu.mx

**Keywords:** *Botrytis cinerea*, antifungal activity, copper-oxide, nanoparticles, green synthesis

## Abstract

**Background/Objectives:** *Botrytis cinerea* is a necrotrophic fungal plant pathogen responsible for the gray mold disease, affecting several crops of economic importance worldwide. The primary line of control for the disease in the field and post-harvest fruits includes the application of fungicides. However, the emergence of fungal populations resistant to one or more fungicides has increased their application and diminished their effectiveness. Looking at new control strategies, metallic nanoparticles have appeared as a promising alternative for disease treatment. Green-synthesized copper oxide nanoparticles (CuONPs) are considered a feasible alternative, aiming to reduce the generation of environmentally toxic waste through chemical methods. **Methods:** In this work, CuONPs biosynthesized using the supernatant of *Trichoderma asperellum* and *Trichoderma ghanense* were evaluated to determine their antifungal activity against *B. cinerea*. **Results:** Four different formulations of CuONPs were obtained: Ta1, Ta2, Tg1, and Tg2. All formulations displayed antifungal properties, with Tg2 being the most effective and having a high potential in controlling the phytopathogen. CuONPs in the Tg2 formulation were quasi-spherical, ranging in size from 1 to 2.7 nm. **Conclusions:** Furthermore, Tg2 demonstrated greater efficacy than the copper-based commercial fungicide NORDOX^®^ 75W, which showed no inhibitory effect on *B. cinerea* mycelial growth. In summary, the CuONPs reported in this work offer a sustainable and effective alternative for managing the gray mold disease.

## 1. Introduction

The phytopathogen *Botrytis cinerea* is an Ascomycota fungus with a necrotrophic lifestyle that affects many plants of economic importance, causing gray mold disease [1]. This fungus is the second most dangerous plant pathogen in fresh fruits and vegetables, with annual losses exceeding USD 100 billion worldwide [2]. Controlling the phytopathogen requires an integrated strategy that includes removing diseased plant parts, using fungicides, proper irrigation and fertilization, and humidity regulation, inter alia. Repeated application of fungicides remains necessary to manage the phytopathogen; however, it has resulted in *B. cinerea* acquiring increased resistance to one or more commercial fungicides [3].

Within the group of metal-based fungicides commonly applied in vineyards to protect grapevines from fungal infections, the Bordeaux mixture (CuSO_4_) stands out as one of the most widely used. This copper-based fungicide, composed of a mixture of quicklime and copper sulfate, has been applied for decades as an effective measure against diseases such as downy mildew. Typically, between 400 and 800 kg per hectare of Bordeaux mixture, containing approximately 2.55 g/L of copper, is sprayed multiple times throughout the grape-growing season to ensure adequate protection [4,5,6,7]. However, the excessive application of the Bordeaux mixture has been related to the cause of environmental pollution and the emergence of resistant phytopathogens against these products [8,9].

Nanomaterials have emerged as a promising new control strategy to control harmful microorganisms. Metallic nanoparticles have shown high potential as an alternative to conventional fungicides and/or adjuvants in combating fungicide resistance [10,11]. Nanoparticles of silver (Ag), zinc oxide (ZnO), and copper oxide (CuO) are mostly investigated for agricultural applications; they can be obtained through chemical, physical, and biological methods. However, physical and chemical processes have shown limitations, such as high cost and the generation of potentially toxic waste [12,13]. On the other hand, biosynthesis using biological resources such as fungi or plants has emerged as a simple, large-scale, cost-effective, and environmentally friendly alternative [14]. Using fungal species to synthesize CuONPs and other metallic nanoparticles has been extensively investigated [15]. Among these fungal species, those of the genus *Trichoderma* stand out because of their high production of antifungal metabolites and lytic enzymes [16,17]. These fungi are widely distributed in soil, exhibit high metal tolerance, and have proven to be excellent biological control agents and plant growth promoters [14].

Previous studies have demonstrated the successful biosynthesis of copper oxide nanoparticles using *Trichoderma* extracts or supernatants. For instance, Saravanakumar and colleagues (2018) synthesized spherical CuONPs ranging in size from 10 to 190 nm, with an average diameter of 110 nm, using cell-free extracts of *Trichoderma asperellum* [18]. Additionally, the antifungal activity of CuONPs synthesized using cell filtrates of *Trichoderma harzianum* and *T. asperellum* has been explored. These nanoparticles exhibited significant inhibitory activity against the phytopathogens *Alternaria alternata*, *Pyricularia oryzae,* and *Aspergillus niger*, highlighting their potential for agricultural applications [19,20]

In a recent study, the antifungal activity of sulfur and copper nanoparticles against *B. cinerea* and *Sclerotinia sclerotium* was evaluated in vitro, comparing their antifungal effectiveness with the fungicide Topsin-M 70 WP. Copper nanoparticles showed significantly more effectiveness, achieving a notable reduction in the mycelial growth of those phytopathogens, in contrast to the reference fungicide, which required higher concentrations to achieve similar results [21].

In this study, *Trichoderma* supernatants were used to synthesize copper oxide nanoparticles. *Trichoderma asperellum* and *Trichoderma ghanense* were selected by preliminary laboratory data, revealing distinct antifungal profiles. Two supernatants (S1 and S2) were obtained under different culture conditions to explore their effect on nanoparticle antifungal characteristics. Four formulations were obtained, showing antifungal activity against *B. cinerea*, the causal agent of the gray mold disease. The results obtained are promising, highlighting the potential application of the formulations as an efficient alternative for controlling this phytopathogen.

## 2. Results

### 2.1. Identification of B. cinerea H13

**Figure 1:**
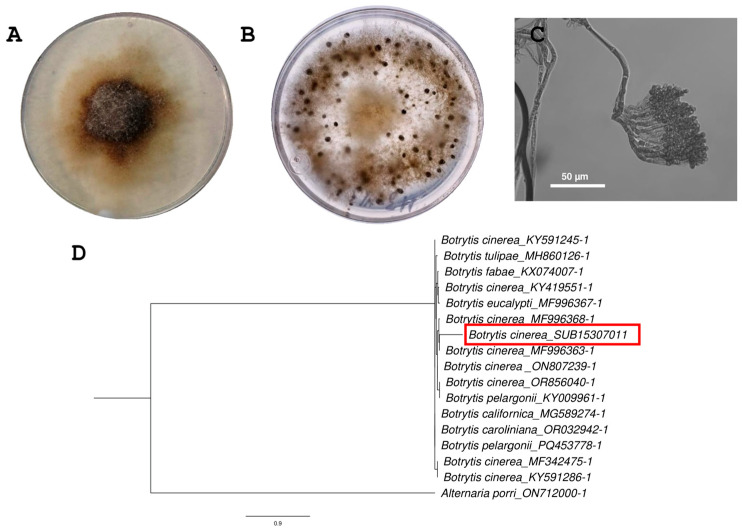
*B. cinerea* isolated from a vineyard in Ensenada, México. (**A**) Morphological characteristics on Minimal Medium. (**B**) Morphological characteristics on PDA medium. (**C**) Branched conidiophores. (**D**) Phylogram based on the sequence of the ITS regions of the isolated SUB15307011 strain and species of the *Botrytis* genus. The strain isolated from the field was located within the *B. cinerea* cluster and is marked in red.

The fungal isolate M3-H13 exhibited a characteristic colonial morphology of *B. cinerea*. It displayed a brown-white mycelium in Minimal Medium (MM) (Figure 1A) and grayish-white mycelium in PDA medium, which darkened over time and developed sclerotia (Figure 1B). Microscope observations revealed structures such as long, branched conidiophores with clusters of conidia, and the conidia showed a characteristic ovoid shape (Figure 1C). To confirm the taxonomic identity of the strain *B. cinerea* H13 (SUB15307011), the phylogenetic analysis of the ITS regions was performed. The phylogram (Figure 1D) shows that strain H13 (SUB15307011) clusters within a well-supported monophyletic clade together with other *Botrytis cinerea* strains (MF996338.1, MF996363.1, ON8072339.1) with an SH-aLRT support value of 0.964.

### 2.2. Plant Virulence Assay

**Figure 2:**
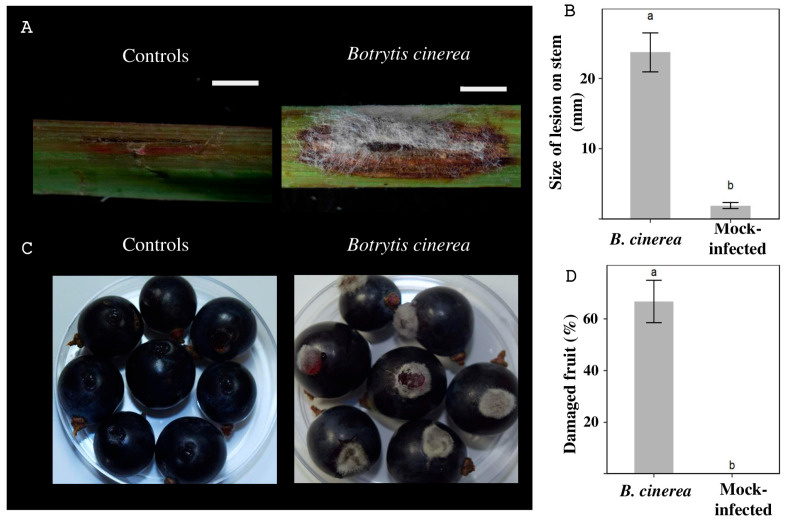
Pathogenicity test of the *B. cinerea* H13 isolate on *Vitis vinifera*, variant Tempranillo, after 96 h. (**A**) Infection on stem, scale bars = 2 mm. (**B**) Area with lesions on stems. (**C**) Infection on fruits. (**D**) Percentage of fruits with damage. Bars with different letters (a, b) differ significantly (*p* < 0.05).

To evaluate the pathogenicity of *B. cinerea* H13 isolate, disease assays were conducted using fruits, and stem segments of *Vitis vinifera* tissues (Tempranillo variety) from the same collection site. Wilting was observed in the stem on the area near the inoculation point, which extended towards the edges (Figure 2A), resulting in an average lesion size of 23.69 mm (Figure 2B) after 96 h. Also, the fungus colonized the fruits, causing softening, followed by collapsed tissue (Figure 2C) after 96 h. Notably, these severe symptoms developed in a substantial 66.66% of the inoculated fruits (Figure 2D).

### 2.3. Copper Oxide Nanoparticles Characterization

**Figure 3:**
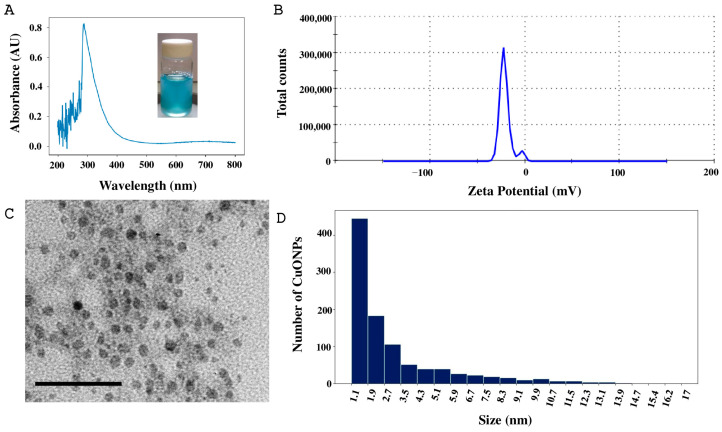
Characterization of CuONPs-Ta1. (**A**) UV–Vis absorbance curve. (**B**) Determination of zeta potential. (**C**) Micrograph of the CuONPs, scale bar = 50 nm. (**D**) Size distribution histogram of CuONPs.

Four different formulations of copper oxide nanoparticles (CuONPs) were synthesized, identified as Ta1, Ta2, Tg1, and Tg2. The synthesis of CuONPs was indicated by the formation of a blue precipitate. Characterization of the Ta1 formulation with UV–visible spectroscopy revealed a distinct maximum absorption peak at 286 nm (Figure 3A), confirming the presence of the compound. The hydrodynamic size was 215.4 nm, and the surface charge (zeta potential) was −26.5 mV (Figure 3B). Moreover, nanoparticles were analyzed by TEM to examine their shape and size, revealing a quasi-spherical shape (Figure 3C). From TEM images, a total of 1000 nanoparticles were measured; sizes were from 1 to 14.7 nm and most of them fell within the 1 to 3 nm range (Figure 3D).

**Figure 4:**
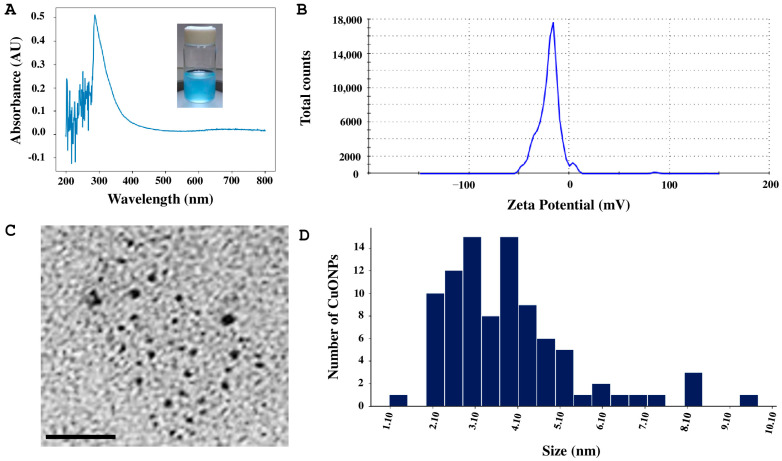
Characterization of CuONPs-Ta2. (**A**) UV–Vis absorbance curve. (**B**) Determination of zeta potential. (**C**) Micrograph of the copper oxide nanoparticles, scale bar = 50 nm. (**D**) Size distribution histogram of CuONPs; a total of 1000 nanoparticles were measured.

The formulation CuONPs-Ta2 was also analyzed, which showed a maximum peak value of 288 nm in UV–Vis spectroscopy (Figure 4A), while the hydrodynamic size of CuONPs was 298.7 nm and the surface charge (zeta potential) was −19.2 mV (Figure 4B). Additionally, small nanoparticles were observed in TEM analysis (Figure 4C). These were identified as quasi-spherical nanoparticles, the majority with sizes ranging from 1 to 5 nm (Figure 4D).

**Figure 5:**
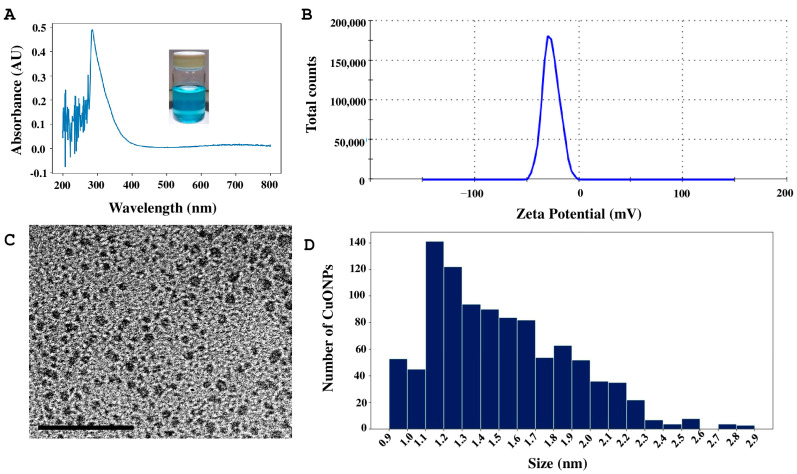
Characterization of CuONPs-Tg1. (**A**) UV–Vis absorbance curve. (**B**) Determination of zeta potential. (**C**) Micrograph of the copper oxide nanoparticles, scale bar = 50 nm. (**D**) Size distribution histogram of CuONPs.

Another formulation, CuONPs-Tg1, showed an absorbance with a maximum peak value of 286 nm in UV–Vis spectroscopy (Figure 5A). The hydrodynamic diameter analysis of the CuONPs was 215.4 nm, and the zeta potential was −26.6 mV (Figure 5B). According to the TEM analysis, this formulation is composed of nanoparticles with quasi-spherical shape (Figure 5C) with a size range of 1 to 2.9 nm (Figure 5D).

**Figure 6:**
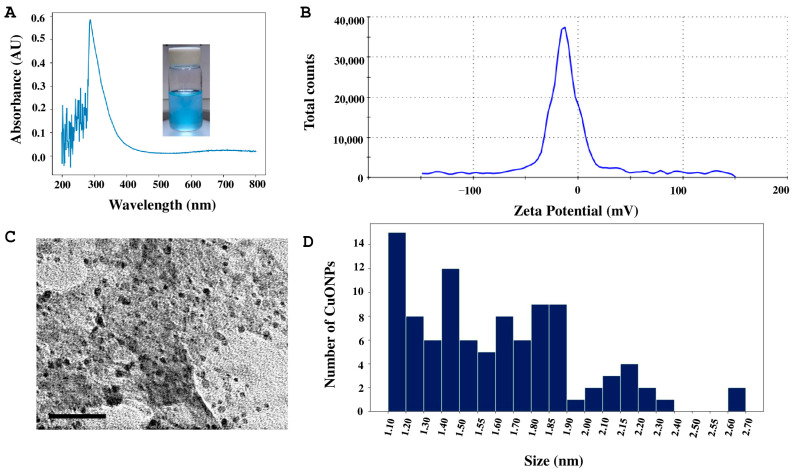
Characterization of CuONPs-Tg2. (**A**) UV–Vis absorbance curve. (**B**) Determination of zeta potential. (**C**) Micrograph of the copper oxide nanoparticles, scale bar = 50 nm. (**D**) Size distribution histogram of CuONPs.

The CuONPs-Tg2 was also characterized; nanoparticles showed a maximum peak value of 288 nm in UV–Visible spectroscopy consistent across all formulations (Figure 6A). The hydrodynamic size was 193.4 and the zeta potential was −7.88 mV (Figure 6B). TEM analysis shows presence of nanoparticles with a spherical form and are inside a matrix by supernatant components (Figure 6C). The nanoparticle sizes range from 1 to 2.7 nm (Figure 6D).

### 2.4. Antifungal Properties of CuONPs Against B. cinerea CDBBH1556

**Figure 7:**
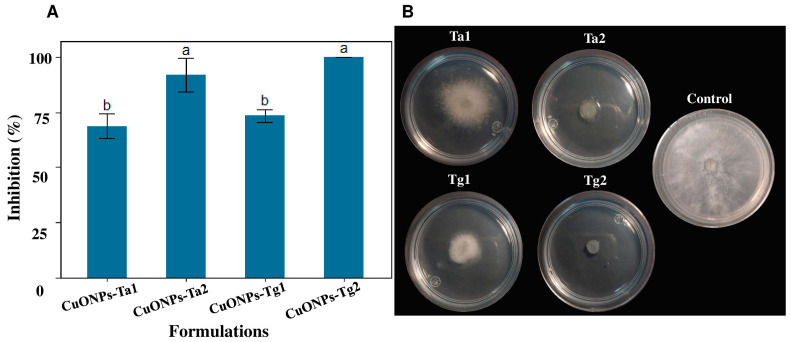
Inhibition of *B. cinerea* CDBBH1556 growth with different treatments at the same concentration of 160 μg/mL. (**A**) Results are expressed as the mean ± SD (n = 3). Bars with different letters indicate statistically significant differences (*p* < 0.0001) (one-way ANOVA with Tukey’s test). (**B**) Representative photographs of the inhibition of the mycelial growth.

Antifungal activity of the CuONPs was evaluated with the determination of the inhibition on the radial growth of *B. cinerea.* The formulation CuONPs-Tg2 presented a high level of inhibition causing 100% at 160 μg/mL (Figure 7A). In contrast, the formulations CuONPs-Ta1, CuONPs-Ta2, and CuONPs-Tg1 exhibited 68.6%, 91.6%, 73.33% of inhibition, respectively (Figure 7A). Results show that all formulations caused inhibitory activity to the radial growth of *B. cinerea* (Figure 7B).

By increasing the concentration to 170 μg/mL, the inhibitory activity of the other formulations increased up to 96.6% for CuONPs-Ta1, and 98.33 for CuONPs-Ta2 and Tg1 (Table 1).

### 2.5. Antifungal Properties of CuONPs Against B. cinerea Isolated from the Field

**Figure 8:**
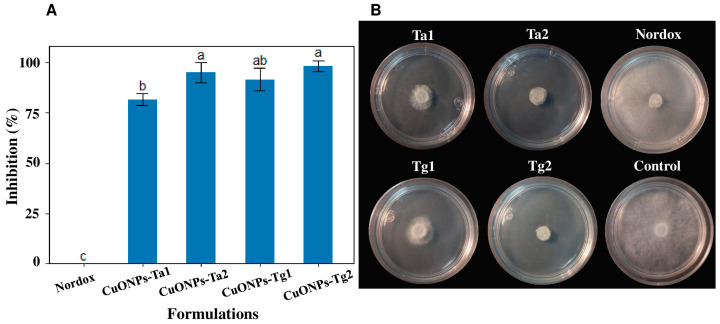
The inhibition of *B. cinerea* H13 (SUB15307011) growth with different treatments at the same concentration of 170 μg/mL. (**A**) Results are expressed as the mean ± SD (n = 3). Bars with different letters indicate statistically significant differences (*p* < 0.0001) (one-way ANOVA with Tukey’s test). (**B**) Representative photographs of the inhibition of the mycelial growth.

The radial growth of the *B. cinerea* isolate H13 (SUB15307011) was inhibited under different antifungal treatments. At a Minimum Inhibitory Concentration (MIC) of 170 μg/mL, the formulation CuONPs-Tg2 showed the highest inhibition with 98.33%, followed by CuONPs-Ta2 with 95% inhibition, CuONPs-Tg1 with 91.66%, and CuONPs-Ta1 with 81.66% (Figure 8A). In contrast, no fungal inhibition was observed in the media poisoned with commercial fungicide, with NORDOX^®^ 75W (Figure 8B).

### 2.6. Antifungal Properties of Trichoderma Supernatants Against B. cinerea Isolated from the Field

**Figure 9:**
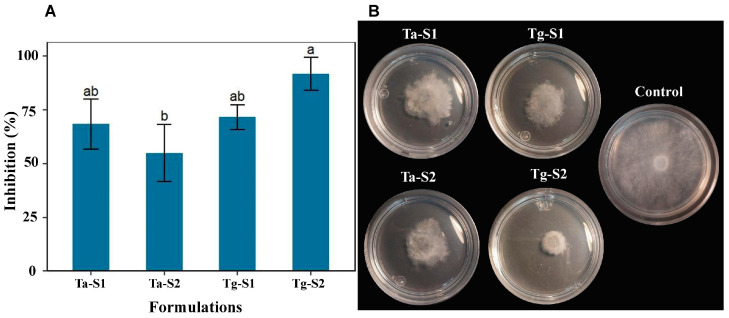
The inhibition of *B. cinerea* H13 (SUB15307011) growth with different supernatant treatments at the same concentration of 42%. (**A**) Results are expressed as the mean ± SD (n = 3). Bars with different letters indicate statistically significant differences (*p* < 0.0001) (one-way ANOVA with Tukey’s test). (**B**) Representative photographs of the inhibition of the mycelial growth.

Poisoned assays were conducted to compare the inhibitory activity of the supernatants and CuONPs by incorporating each supernatant at 42% into PDA, matching the concentration used in the nanoparticle formulation. According to the results, the greatest inhibition was observed in the Tg-S2 with 86.7%; subsequently, Tg-S1 showed 71.7%; Ta-S1, 68.33%; and Ta-S2, 53.33 (Figure 9A). Even though radial colony expansion was limited, aerial mycelium continued to develop (Figure 9B).

### 2.7. Chitinase Assays

**Figure 10:**
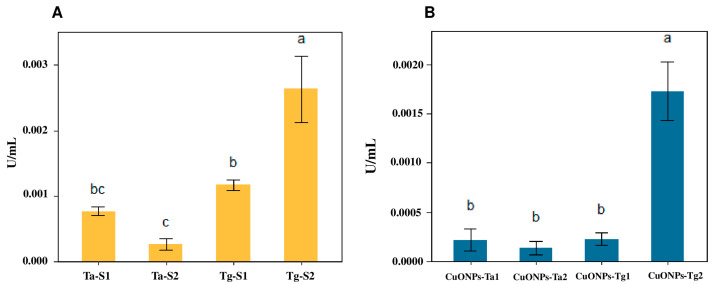
Chitinase activity determination. (**A**) Supernatants used as reducing agents. (**B**) CuONPs formulations. Results are expressed as the mean ± SD (n = 3). Bars with different letters indicate statistically significant differences (*p* < 0.0001) (one-way ANOVA with Tukey’s test).

The chitinase activity was determined in the supernatants and the CuONPs formulations. The supernatants showed chitinase activity (Figure 10A). The supernatant of *T. ghanense* (Tg-S2) had the highest value of 0.0026 U/mL, followed by Tg-S1 with 0.0011 U/mL. On the other hand, the supernatants of *T. asperellum* displayed less chitinase activity with 0.0008 U/mL for Ta-S1 and 0.0002 U/mL for Ta-S2. Chitinase activity in nanoparticles formulations (Figure 10B) indicates that the highest value corresponds to CuONPs-Tg2 formulation, with 0.0017 U/mL, while the other formulations exhibit values close to 0.0002 U/mL.

### 2.8. Biocompatibility Evaluation

**Figure 11:**
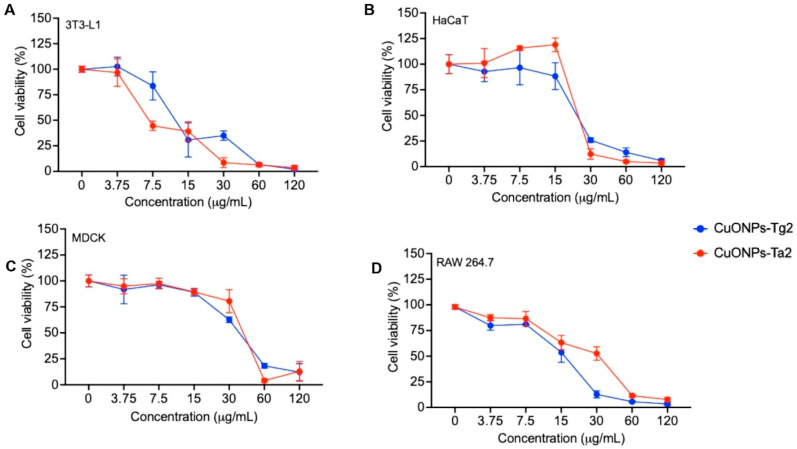
Cytotoxicity assays of CuONPs in cell lines from (**A**) fibroblasts 3T3-L1, (**B**) keratinocytes HaCaT, (**C**) kidney MDCK, and (**D**) macrophages RAW 264.7.

Cytotoxicity assays of CuONPs were performed on four cell lines: two skin epithelial models (3T3-L1 fibroblasts and HaCaT keratinocytes), a renal model (MDCK), and macrophages (RAW 264.7) representing the innate immune system. Assays were carried out using the formulations with the highest fungal inhibition activity (CuONPs-Tg2 with 98.33% and CuONPs-Ta2 with 95%). As shown in Figure 11, both CuONPs formulations were cytotoxic in all the cell lines. CuONPs-Tg2 proved most toxic across the board, except in 3T3-L1 fibroblasts, which were slightly more susceptible to CuONPs-Ta2, as confirmed by the IC50 values in Table 2. Sensitivity ranked as follows: MDCK > macrophages > keratinocytes > fibroblasts.

**Figure 12:**
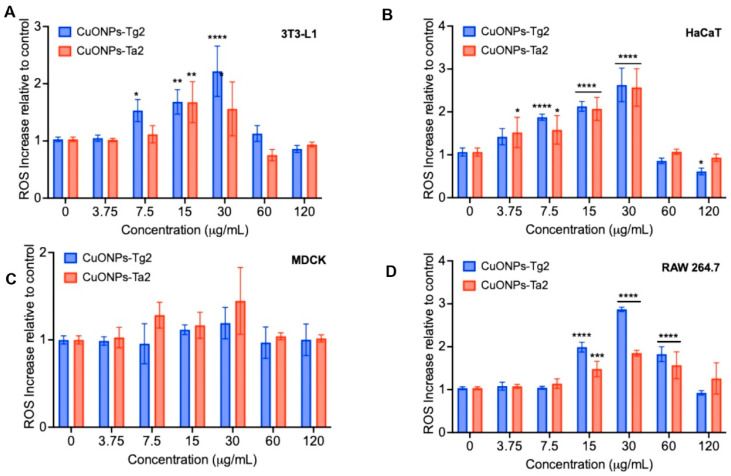
Cellular production of ROS in (**A**) fibroblasts 3T3-L1, (**B**) keratinocytes HaCaT, (**C**) kidney MDCK, and (**D**) macrophages RAW 264.7, subjected to varying concentrations of CuONPs. Results are presented as the mean ± SD (n = 3). * *p* < 0.05; ** *p* < 0.01; *** *p* < 0.001; **** *p* < 0.0001 using with two-way ANOVA with a Dunnett’s multiple comparisons test.

One of the main toxicity mechanisms of metal and metal-oxide nanoparticles is the overproduction of ROS, leading to oxidative stress. Accordingly, ROS generation was measured in each cell line after exposure to CuONPs. As shown in Figure 12, revealing a pattern of susceptibility that correlated with cytotoxicity, CuONPs-Ta2 triggered ROS increases from 15 µg/mL onward in nearly every cell line except MDCK renal cells. In contrast, CuONPs-Tg2 induced an even greater ROS surge, starting at 7.5 µg/mL, in all evaluated cells, with MDCK remaining the most resistant to oxidative stress.

**Figure 13:**
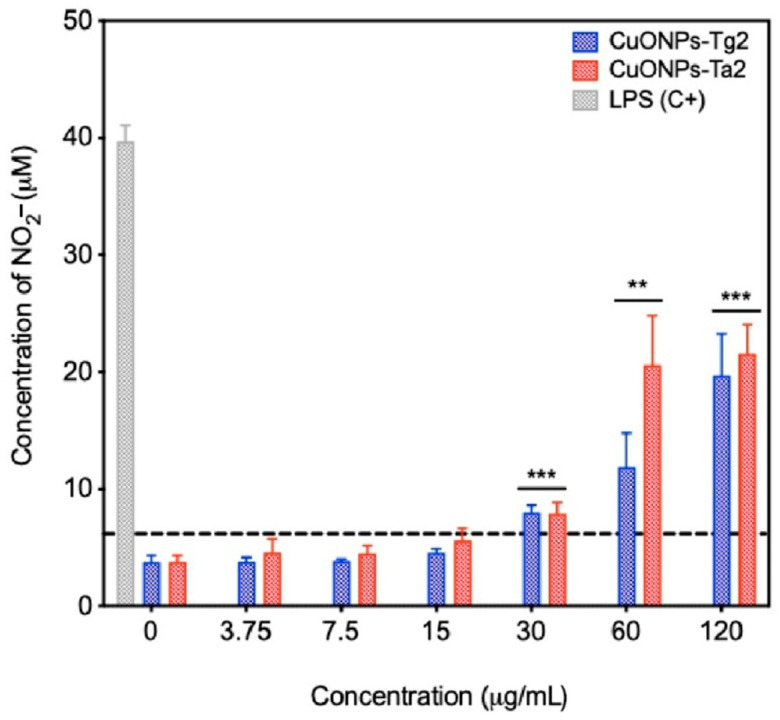
Nitrite levels produced by macrophages RAW 264.7 treated with CuONPs. Results are presented as the mean ± SD (n = 3). ** *p* < 0.01; *** *p* < 0.001; two-way ANOVA with a Dunnett’s multiple comparisons test. The dotted line denotes the specified threshold.

Nitric oxide (NO) acts as an essential second messenger in various cellular signaling pathways, particularly those involved in immune responses. Excessive NO production is linked to inflammatory processes, since NO can react with oxygen to generate highly cytotoxic ROS. Furthermore, metallic nanoparticles have been reported to stimulate NO synthesis, leading to cell death or inflammatory responses. As shown in Figure 13, the Ta2 and Tg2 CuONPs formulations at concentrations of 30 µg/mL and above elicited increased NO production, with CuONPs-Ta2 generating the highest levels. Importantly, the NO concentrations induced by CuONPs never approached those measured in the LPs positive control.

## 3. Discussion

Copper oxide nanoparticles (CuONPs) could be useful in various applications, including agriculture. Studies have shown that CuONPs enhance plant growth and development by improving photosynthetic efficiency, nutrient absorption, and root growth [22]. In addition, several studies have reported the high antifungal activity of CuONPs synthesized by green methods using fungal extracts [20,23,24,25,26]. Among fungi, *Trichoderma* species have been successfully employed in the green synthesis of nanoparticles, making them valuable tools in sustainable plant protection strategies. *Trichoderma* species have shown effectiveness as biocontrol agents attributed to multiple mechanisms, including nutrient competition, mycoparasitism, antibiotic production, the production of lytic enzymes, and the promotion of plant defense systems [27,28]. To harness the broad spectrum of secondary metabolites and enzymes produced by *Trichoderma*, in this work, two different supernatants were obtained: supernatant 1 (S1) from culturing the fungi (*T. ghanense* C4B or *T. asperellum* T8a) in PDB; and supernatant (S2) from incubating the obtained fungal biomass of the *Trichoderma* species mentioned in deionized water. During the synthesis process, the supernatant acts as reducing and capping agent [29]. In the synthesis reaction, different incubation conditions such as temperature, metal precursor concentration, pH, and culture medium can result in nanoparticles with different physicochemical characteristics [30].

In this study, the synthesis protocol was carried out at room temperature (24 °C). It has been reported that temperature influences the rate of nanoparticles synthesis. Several studies indicate that faster synthesis rates are achieved at higher temperatures. However, the size and stability of the nanoparticles may be compromised. In this work, good stability was observed in all formulations synthesized at room temperature (24 °C), consistent with the study by Shahzad et al. (2019), who reported smaller nanoparticles with greater stability at temperatures close to 20 °C [31]. As previously mentioned, the pH is another important aspect to consider during the synthesis reaction, as the conformation of nitrate reductase enzymes can be altered by the proton concentration in the reaction medium, affecting the morphology and size of nanoparticles [32]. In this study, the synthesis reaction was adjusted to an alkaline pH: synthesis with S1 were conducted at pH of 10, while those with S2 were performed at pH of 8. This approach is based on studies suggesting that at higher pH, there is greater competition between protons and metal ions to establish bonds with negatively charged regions, resulting in more successful synthesis of nanoparticles [33,34]. Also, it is important to consider the metal precursor concentration. In this case, 15 mM was used to avoid an excessive amount of metal ions, as higher concentrations have been reported to cause the formation of very large nanoparticles with irregular morphology due to competition between the ions and the components in the fungal supernatant [30].

In addition to the antimicrobial properties of CuONPs, the *Trichoderma* genus secretes an enzymatic cocktail that includes chitinases, β-glucanases and proteases [17,35,36,37]. Chitinases are the most abundantly produced mycoparasitic enzymes by this fungal genus. The enzymes are glucosyl hydrolases with low molecular weight, typically ranging from 20 to 90 kDa [35].

During the synthesis protocol, maintaining the stability of enzymatic activity in the supernatant was crucial to maximizing the antifungal properties in the formulation. Results from the enzymatic activity indicate that the enzymatic activity decreases in all formulations; however, the CuONPs-Tg2 formulation retained higher enzymatic activity with 0.0017 U/mL, while the other formulations exhibited values close to 0.0002 U/mL. In the case of the TgS2 supernatant, the reduction was only about 35%, whereas for the TaS2 supernatant, a reduction of up to 55% was observed. These results indicate that a significant proportion of the enzymatic activity was preserved during nanoparticles formulation.

*Trichoderma* spp. are recognized as prolific producers of secondary metabolites with diverse biological functions. The recently published genomes of several *Trichoderma* species revealed an extensive repertoire of genes potentially involved in secondary metabolism, with differences between species [38,39]. These differences in metabolic capacity may contribute to the variability observed in the biochemical composition of culture supernatants, which can, in turn, influence enzyme stability and activity retention.

The copper oxide nanoparticles (CuONPs) produced in the four formulations exhibited a small size and quasi-spherical shape, like the biosynthesized copper nanoparticles reported by the authors of [40] using supernatant of *T. harzianum*. Regarding size, in this study, small nanoparticles ranging from 1 to 3 nm were obtained, which aligns with previous studies [26], where biosynthesized nanoparticles with *Trichoderma* sp. had an average size of 5.8 nm.

Nanoparticle size is an aspect that has been linked to its mechanism of action. Some studies describe that their impact on fungi can occur in hyphae and spores, as it has been reported that fungi treated with metal nanoparticles exhibit hyphal deformations, related to damage to the cell wall [41]. Smaller nanoparticles can enter the cell and cause intracellular damage, including mitochondrial fragmentation, ribosome depolymerization, and chromatin damage, leading to the formation of multivesicular bodies. Furthermore, several studies suggest that the formation and accumulation of reactive oxygen species (ROS) in cells is the primary mechanism by which nanoparticles act against pathogenic microorganisms [41,42].

In this study, the Minimum Inhibitory Concentration (MIC) was determined for the four nanoparticle formulations obtained. The best results were observed with the CuONPs-Tg2 formulation, which showed an MIC of 160 µg/mL for the *B. cinerea* CDBBH1556 strain, while a concentration of 170 µg/mL was required for field-isolated *B. cinerea*. Few studies have evaluated the exposure of *B. cinerea* to biosynthesized CuONPs. However, the results obtained in this work align with those reported by the authors of [43], who reported an MIC of 160 µg/mL to inhibit the phytopathogen using chemically synthesized CuONPs. Nevertheless, in their case, the nature of the nanoparticles used may compromise environmental balance and pose health risks. On the other hand, the use of biosynthesized CuONPs to inhibit the growth of other phytopathogenic fungi has been described. Recently, it was reported that nanoparticles biosynthesized with the fungus *T. asperellum* required up to 200 µg/mL to inhibit the growth of *Alternaria brasicae* [25], the same concentration reported to inhibit the phytopathogen *Fusarium solani* [24]. The antifungal activity of the CuONPs-Tg2 formulation was compared to the commercial fungicide NORDOX^®^ 75W (cuprous oxide) at the same concentration (170 µg/mL). The antifungal effect of the CuONPs formulations was higher than the commercial fungicide; part of this effect may also be due to the action of residual bioactive compounds present in the supernatants. Therefore, the contributions of enzymatic and copper-based mechanisms cannot be completely separated.

To determine the CuONPs biocompatibility, their cytotoxic effect was evaluated in four cellular models: skin epithelial models (3T3-L1 fibroblasts and HaCaT keratinocytes), a renal model (MDCK), and macrophages (RAW 264.7) representing the innate immune system. Cell viability depends on multiple factors, such as cell type, concentration, exposure time, and the physicochemical properties of the nanoparticles [44]. In this study, the CuONPs-Tg2 formulation showed a higher induction of nitrite (NO) production in macrophages starting at 30 µg/mL and reactive oxygen species (ROS) from 7.5 µg/mL, while the CuONPs-Ta2 did so starting at 15 µg/mL. The toxicity of CuONPs varied according to the cell line. In MDCK cells, which overexpress high-affinity copper transporters, a drastic loss of cell viability was observed, starting at 30 µg/mL. In this sense, previous studies have shown that exposure above 20 µg/mL reduce the viability of these cells by decreasing metabolic activity, depolarizing the mitochondrial membrana potential, and arresting the cell cycle in the G2/M phase, leading to increased cell death [45]. In macrophages (RAW 264.7), cell viability decreased from 15 µg/mL. Gupta et al. reported that CuONPs are rapidly internalized by the cells and accumulate in lysosomes, causing non-apoptotic cell death [46]. This effect has been reduced with the use of copper chelating agents, suggesting that the release of Cu^2^+ ions is a key mechanism of cytotoxicity [47]. A similar effect has been observed in in vitro studies with mouse embryonic fibroblasts (3T3-L1), where starch-coated copper nanoparticles showed cytotoxicity only at concentrations significantly higher than those required for free cupric ions [47].

Interestingly, a recent study comparing corrective treatments for symptomatic grapevines plants reported that the application of fungicide NORDOX^®^ 75W resulted in a higher accumulation of copper in plant tissues compared to plants treated with CuONPs obtained via green synthesis [20]. These findings suggest that biosynthesized CuONPs could serve as an alternative corrective treatment, reducing copper accumulation in grape tissues and mitigating potential environmental and health risks associated with conventional copper-based fungicides.

It is important to note that, unlike copper oxide nanoparticles, copper salt-based fungicides release a greater amount of free Cu^2+^ ions, which can increase their toxicity due to the greater availability of these ions. Therefore, by using CuONPs in the field, the release of free Cu^2+^ ions would be significantly reduced. However, due to their potential toxicity, these materials should be used appropriately to minimize risks to human health. Finally, these results provide compelling evidence that the CuONPs-Tg2 formulation is highly effective against *B. cinerea*, including the strain isolated from a vineyard. Moreover, its effectiveness, comparable to the synthetic nanoparticles, combined with its lower environmental impact, highlights the advantage of the biosynthesized nanoparticles reported in this study.

## 4. Materials and Methods

### 4.1. Strains, Media and Growth Conditions

The strains *T. asperellum* T8A, *T. ghanense* C4B, and *B. cinerea* CDBBH1556 were obtained from the strain collection of the Department of Microbiology at CICESE. *B. cinerea* H13 (SUB15307011) was isolated from symptomatic *Vitis vinifera* plants (Tempranillo variety) in a vineyard located in Ensenada, Baja California, México (latitude: 31.98494, longitude: −116.6465755). *T. ghanense* and *T. asperellum* were cultured in Petri dishes containing Potato Dextrose Agar (PDA) at 25 °C for five days to obtain conidia. Similarly, *B. cinerea* strains were grown in PDA at 25 °C for seven days to harvest conidia.

### 4.2. Morphological and Molecular Identification of B. cinerea H13

The fungal isolate *B. cinerea* H13 (SUB15307011) was morphologically and molecularly identified. For morphological characterization, the fungus was grown in Potato Dextrose Agar (PDA) and Minimal Medium (MM) at 25 °C for seven days. The identification of microscopic structures was carried out using Differential Interference Contrast (DIC) with a Nikon Eclipse TLF series microscope (Nikon Corporation, Tokyo, Japan). For the molecular identification, the fungal isolates’ DNA extraction was performed using a QIAGEN DNA Plant Tissue Kit (QIAGEN N.V., Hilden, Germany). PCR amplifications of the internal transcribed spacer (ITS) were carried out using the primers ITS5 (5′-GGAAGTAAAAGTCGTAACAAGG-3′) and ITS4 (5′-TCCTCCGCTTATTGATATGC-3′) [48]. The amplicons were purified and then sequenced. Multiple alignments were carried out with MAFFT software (version 7) and phylogenetic inference with the maximum likelihood (ML) method in PhyML, using Smart Model Selection (SMS) to choose the best evolutionary model. The statistic support was evaluated through Shimodara–Hasegawa-like approximate likehood ratio test (SH-aLRT).

### 4.3. Plant Virulence Assay for B. cinerea H13

*Vitis vinifera* tissues (Tempranillo variety) were used to conduct virulence assays. Fruits and stem segments of 10 cm in length were used. Plant tissues were inoculated with *B. cinerea* H13 mycelial plugs of 5 mm in diameter. The plant tissues were first washed with tap water to remove dust and then washed with abundant distilled water. Additionally, fruits were washed with 10% hypochlorite for disinfection. Afterward, a sterile scalpel was used to create a 5 mm lesion on the plant tissues, and then the inoculum was placed to ensure direct contact of the mycelium and the plant. The experiment was performed at room temperature under 12 h light–dark cycle conditions. Symptoms were monitored every 24 h. The assay was performed three times, each time in triplicate.

### 4.4. Supernatants Obtention

Fungal supernatants for copper oxide nanoparticles (CuONPs) biosynthesis were obtained according to [18], with some modifications. *T. asperellum* T8A and *T. ghanense* C4B were cultured in Potato Dextrose Broth (PDB) medium with a concentration of 1 × 10^6^ conidia and constant agitation at 130 rpm for 5 days at 25 °C. After the incubation time, the supernatants (S1) were separated and filtered with a 0.22 µm nitrocellulose membrane and refrigerated until use. The biomass from the liquid cultures was washed with deionized water and weighed. Thereafter, the biomass was transferred to flasks containing sterile deionized water at a 1:10 proportion (biomass: water) and incubated for three days at 25 °C with agitation at 130 rpm. The resulting supernatants (S2) were obtained and filtered with 0.22 µm nitrocellulose membrane.

### 4.5. Biosynthesis and Characterization of CuONPs

Nanoparticles were synthesized using pentahydrated copper (II) sulfate (CuSO_4_·5H_2_O) at 15 mM, and 0.1 M sodium hydroxide (NaOH) solution was added for pH 10 adjustment for S1 and pH 8 for S2. The pH gradually changed until the color of the reaction mixture changed from clear to light blue. The reaction mixture was prepared at a 1:2 proportion (supernatant: copper sulfate) and incubated at 24 °C for 24 h. Following the reaction mechanism described by [25], the theorical yield of CuONPs was estimated based on the stoichiometric ratios of the reaction.

The characterization of synthesized nanoparticles was performed using ultraviolet–visible spectrophotometry (UV–Vis), dynamic light (DLS), and transmission electron microscopy (TEM). UV–Vis characterization was performed with a Perkin Elmer Precisely UV–VIS Lambda/25 spectrophotometer (PerkinElmer Inc., Waltham, MA, USA), and measurements were taken in the 200–700 nm range. The hydrodynamic diameter and zeta potential (Z-potential) of the CuONPs, and DLS measurements were conducted with a Malvern Zetasizer Nano ZS90 (Malvern Panalytical Inc., Westborough, MA, USA).

The morphology and size of the CuONPs was determined using a Hitachi H7500 electron transmission microscope (Hitachi Ltd., Tokyo, Japan). Samples were deposited onto a carbon-coated grid (200 mesh copper grid) and allowed to dry at room temperature. Finally, they were analyzed at 80 and 100 keV, and images were captured. Nanoparticle size was determined using ImageJ version 1.5 software (free for Windows 1.8.0_172).

### 4.6. Assessment of Antifungal Properties of CuONPs Against B. cinerea CDBBH1556

To study CuONPs antifungal properties, a poisoned medium assay was conducted by adding the nanoparticle formulations to the PDA culture medium. The concentrations of CuONPs were 130, 140, 150, 160, and 170 µg/mL. The plates were inoculated with 5 mm diameter mycelial plugs of *B. cinerea* CDBBH1556 placed at the center of each plate and incubated at 25 °C. Measurements were taken at 24, 48, and 72 h post-inoculation. All assays were performed in triplicate, and fungal growth on the same culture medium without treatments was used as a control. The experiment concluded when the pathogen colony filled the control plate. The inhibition percentage was calculated using the following equation:

$$IR=\frac{R1-R2}{R1}\times 100$$

where

R1: Radial growth of *B. cinerea* in control plates

R2: Radial growth of *B. cinerea* in treatment plates

### 4.7. Assessment of CuONPs Antifungal Properties Against B. cinerea Isolated from the Field

The inhibition of the *B. cinerea* H13 isolate was evaluated in comparison to the reference strain *B. cinerea* CDBBH1556. Nanoparticle formulation was added to the PDA culture medium to achieve this. Following the previous protocol, the plates were inoculated with 5 mm diameter mycelial plugs at the center of each plate. All experiments were performed in triplicate. As a control, plates with culture medium without treatments were used. Additionally, to compare the activity of nanoparticles with a commercial fungicide, a treatment with NORDOX^®^ 75W (NORDOX Industrier AS, Oslo, Norway) (84.4% Cu_2_O) was included (at 170 μg/mL).

### 4.8. Assessment of Antifungal Properties of Trichoderma Supernatants Against B. cinerea Isolated from the Field

*Trichoderma* supernatants were evaluated to determine the inhibitory activity in *B. cinerea* H13 strain. A poisoned medium assay was carried out. Each supernatant was incorporated into PDA at a concentration of 42%, consistent with that used in the nanoparticle formulation. Plates were inoculated with 5 mm diameter mycelial plugs at the center of each plate. All experiments were conducted in triplicate. Control plates contained only the culture medium without any treatment.

### 4.9. Chitinase Activity Assays

To determine if the formulations of synthesized nanoparticles retained enzymes with chitinase activity from the supernatants, the chitinase activity was determined in both the supernatants and the synthesized nanoparticles. Chitinase assays were performed using a Sigma-Aldrich Chitinase assay Kit (Sigma-Aldrich, St. Louis, MO, USA). The unit definition is as follows: one unit will release 0.1 µmole of p-nitrophenol from the appropriate substrate per minute at pH 4.8 at 37 °C. Calculations were performed following the kit directions.

### 4.10. Biocompatibility Evaluation Assays

#### 4.10.1. Cell Culture

Fibroblasts 3T3-L1 (CL-173), HaCaT, kidney MDCK cells (CCL-34), and Macrophages RAW 264.7 (TIB-71), were obtained from the American Type Culture Collection (ATCC). Fibroblast 3T3-L1 were cultured in Dulbecco’s Modified Eagle’s Medium (DMEM) supplemented with calf serum at 10% *v*/*v*. Meanwhile, other cell lines were cultivated with DMEM enriched with 10% Fetal Bovine Serum (FBS, BIO-B., Estado de Mexico, Mexico), 1% Penicillin streptomycin (Sigma-Aldrich, St. Louis, MO, USA), 1% L-glutamine, and 1.5 g/L sodium bicarbonate. The incubation was until confluence at 37 °C and in a 5% CO_2_ atmosphere.

#### 4.10.2. Cell Viability Assay

Susceptibility of cells to CuONPs was determined using a 96-well plate, seeding 10,000 cells per well. The reduction in the MTT reagent 3-(4,5-dimethylthiazol-2-yl)-2,5-diphenyltetrazolium (Sigma-Aldrich) was used to determine cell viability. In accordance with the manufacturer’s instructions, the cells were incubated at 37 °C and in a 5% CO_2_ atmosphere. After incubation, the cell culture medium was discarded and fresh medium containing varying concentrations of CuONPs (3.75 to 120 µg/mL) was added in a final volume of 100 µL. These concentrations were chosen to include the MIC; however, due to the constraints of the cell-based assay, the highest concentration tested was limited to 120 µg/mL. As a result, this was the highest concentration tested, even though it did not fall within the MIC range observed in fungi. Then, cells were incubated for an additional 24 h at the same conditions. Subsequently, the medium was discarded, and the cells were rinsed three times with 200 µL of PBS 1×. To assess cytotoxicity, the MTT assay was performed. As a positive control, untreated cells were used, while cells treated with 100 µL of 0.5% Triton X-100 in PBS served as the negative control. Absorbance resulting from MTT reduction was measured using a 96-well plate reader (Cytation1, BioTek, Santa Clara, CA, USA). Background absorbance at 690 nm was excluded from the absorbance at 570 nm, which reflects cell viability. All experiments were carried out independently in triplicate, with each experiment containing internal triplicates.

#### 4.10.3. Reactive Oxygen Production

Reactive oxygen species (ROS) production was assessed by flow cytometry. A total of 50,000 cells were seeded into each well of a 24-well plate and incubated for 24 h at 37 °C and 5% CO_2_, in the presence of CuONPs at concentrations ranging from 3.75 to 120 µg/mL. Following incubation, cells were rinsed and exposed to 30 μM of 2′,7′-dichlorofluorescein diacetate for 90 min under the same temperature and CO_2_ conditions. Afterward, the cells were rinsed and complete DMEM was added to each well. Basal ROS levels were determined in untreated cells. Fluorescence measure was performed employing a Varioskan microplate reader (Thermo Scientific, Waltham, MA, USA) with excitation and emission wavelengths of 485 and 530 nm, respectively.

#### 4.10.4. Production of Nitrite by Macrophages

Nitrite production by macrophages following incubation with CuONPs was quantified employed by Griess assay. The oxidation of nitric oxide results in nitrites production, a molecule produced by macrophages as part of the immune response to pathogens and for regulating inflammation and other physiological processes [49]. To detect nitric oxide production, the Griess reaction was performed as follows:

Macrophages were plated into a 96-well plate at a density of 10,000 cells per well and incubated for 24 h at 37 °C and 5% CO_2_ atmosphere. Following the incubation period, various concentrations (ranging from 3.75 to 120 µg/mL) of CuONPs were added, followed by an additional 24 h incubation of CuONPs, followed by an additional 24 h incubation under the same conditions.

Following staining, the culture medium from each well was transferred to a new plate and mixed with 5 mM sodium nitroprusside. The mixture was incubated for 1 h in the dark at 37 °C and 5% CO_2_. Subsequently, 0.1% Griess reagent solution (Sigma-Aldrich, St. Louis, MO, USA) was added to each well and incubated for 15 min at 25 °C in the dark. Absorbance was measured at 540 nm, and nitrite levels were determined by comparison with a standard curve generated using sodium nitrite in the range of 1.67 to 100 µM. All experiments were carried out independently in triplicates, with each condition tested with internal triplicates.

### 4.11. Statistical Analysis

All experiments were performed in both technical and biological triplicates. Results are presented as mean values ± SD. Data were analyzed using R-Study software (version 4.5.0). Statistical significance between treatments means was assessed using either Tukey’s test or Student’s *t*-test. Plots and analyses were performed with Prism GraphPad version 10 (Boston, MA, USA).

## 5. Conclusions

The biological synthesis of CuONPs using the supernatants of *T. ghanense* and *T. asperellum* were performed. In general, small (from 1 to 3 nm), quasi-spherical, and stable nanoparticles were obtained and showed excellent inhibition efficacy against the causal agent of the gray mold disease *B. cinerea*. The results obtained open new perspectives for developing innovative and effective antifungal formulations. The CuONPs-Tg2 formulations showed greater efficacy compared to the other formulations and even outperformed a commercial fungicide, highlighting its potential for field applications as an efficient alternative to control phytopathogens.

## Figures and Tables

**Table 1:** Minimum Inhibitory Concentration (MIC) determination in *B. cinerea* CDBBH1556. Values represent the percentage of inhibition of mycelial growth.

Treatment (CuONPs)	130 μg/mL	140 μg/mL	150 μg/mL	160 μg/mL	170 μg/mL
CuONPs-Ta1	11.66 ± 7.6	16.6 ± 5.7	40 ± 8.6	68.6 ± 5.5	96.66 ± 5.7
CuONPs-Ta2	63.3 ± 5.7	60 ± 10	81.6 ± 10.4	91.6 ± 7.6	98.33 ± 2.8
CuONPs-Tg1	41.66 ± 14.4	58.33 ± 7.6	46.6 ± 7.6	73.33 ± 2.8	98.33 ± 2.8
CuONPs-Tg2	76.6 ± 5.7	75 ± 5	90 ± 8.6	100 ± 0	100 ± 0

**Table 2:** IC_50_ values for CuONPs on cell lines.

Treatment (CuONPs)	IC_50_ μg/mL			
	3T3-L1	HaCaT	MDCK	RAW 264.7
CuONPs-Tg2	14.05 ± 0.881 R^2^: 0.880	10.74 ± 0.936 R^2^: 0.936	7.849 ± 0.955 R^2^: 0.955	8.103 ± 0.948 R^2^: 0.948
CuONPs-Ta2	11.63 ± 0.891 R^2^: 0.891	12.46 ± 0.951 R^2^: 0.951	9.73 ± 0.946 R^2^: 0.946	8.221 ± 0.942 R^2^: 0.943

IC_50_ results are presented as the mean ± SD (n = 3). A dose–response model using sigmoidal log (inhibitor) vs. response curve was selected and fitted to a variable Hill slope.

## Data Availability

Data is contained within the article.

## Abbreviations

The following abbreviations are used in this manuscript:

CuONPs	Copper oxide nanoparticles
MM	Minimal Medium
PDA	Potato Dextrose Agar
ITS	Internal Transcribed Spacers
MIC	Minimum Inhibitory Concentration
ROS	Reactive Oxygen Species
